# Supplementary material for: Identification of the TSSK4 Alternative Spliceosomes and Analysis of the Function of the TSSK4 Protein in Yak (Bos grunniens)
Source: Animals (Basel). 2022 May 27;12(11):1380. doi: 10.3390/ani12111380 (PMC9179852; doi:10.3390/ani12111380)
Supplement: Supplementary file 1 [file animals-12-01380-s001.zip › Supplementary material.pdf]

Identification of the *Tssk4* alternative spliceosomes and analysis of the function of the TSSK4 protein in yaks

Xingdong Wang, Jie Pei, Lin Xiong, Shaoke Guo, Mengli Cao, Yandong Kang, Pengjia Bao, Xiaoyun Wu, Min Chu, Chunnian Liang, Ping Yan* and Xian Guo *

Western blot

1 Protein extraction

The cells were washed 2–3 times with PBS buffer, draining the remaining fluid as much as possible. The tissue was ground to powder in a mortar with liquid nitrogen. Then, an appropriate amount of total cell protein extraction reagent was added to the above cell/tissue powder (a protease inhibitor was added a few minutes before use to make the working concentration 1*) and lysis in the culture plate/flask for 3–5 minutes. The plate/flask was shaken repeatedly during this period to ensure the complete contact of the cells with the reagents. The cells and reagents were scraped off with a cell scraper and collected into a 1.5 mL centrifuge tube. These cells were then incubated over an ice bath for 30 min, during which the cell suspension was pipetted repeatedly to ensure complete cell lysis. The cell suspension was then centrifuged at 12,000 rpm for 5 min at 4°C, and the supernatant was collected, which comprised the total protein solution.

2 Protein concentration quantification

The protein concentration of the samples was determined using the BCA protein concentration assay kit (Solebo., Beijing, China).

3 SDS-PAGE electrophoresis

1) Sample processing

① The loading amount was determined according to the sample concentration ensuring that the total protein loading amount of each sample was 50 μg.

②An appropriate amount of 5× protein loading buffer was added to the protein sample, and bath in boiling water at 95–100℃ for 5 min.

2) Glue making and sample loading

① The separating gel was prepared, shaken well, and poured immediately after adding TEMED. An appropriate amount of water was added slowly to the glue surface to flatten the surface. After about 45 mins, water was drained off the upper layer of the glue and the remaining water was dried with an absorbent paper.

② To prepare stacking gel, the gel was shaken immediately after adding TEMED. The remaining space was filled with the stacking gel and the comb was inserted into the stacking gel.

③The comb was pulled out, and the electrophoresis rack was placed into the elec-trophoresis tank, followed by the addition of the electrophoresis buffer, and the sample to the spotting well.

④The constant voltage electrophoresis was performed at 80 V for the stacking gel and 120 V for the separating gel until the bromophenol blue reached the lower edge of the gel plate.

4 Transfer film

1) The transfer membrane filter paper and PVDF membrane were prepared ac-cordingly The PVDF membrane was activated with methanol before use, and the acti-vation time was 3 min.

2) The transfer film "sandwich" structure was placed in the direction of the positive and negative electrodes. The transfer film sponge, 3 layers of filter paper, PVDF film, glue, 3 layers of filter paper, and the transfer film sponge were arranged in order from the positive electrode to the negative electrode. All the air bubbles were removed from each layer.
3) The membrane was transferred at a constant current of 300 mA, and the transfer time was adjusted according to the molecular weight of the target protein.

5 Antibody Incubation

1) The transferred membrane was added to the blocking solution and blocked at room temperature for 1 h.
2) The blocking solution was removed and the primary antibody diluted with primary antibody diluent was added and incubated at 4°C overnight (primary antibody concentration range 0.5–2ug/ml).
3) The diluted primary antibody was recovered and the membrane was washed three times with TBST for 5 min each.
4) The diluted secondary antibody was added to the secondary antibody diluent, incubated at room temperature for 30 min, and washed with TBST four times at room temperature on a shaker for 5 min each. (Secondary antibody dilution ratio 1:10000)

6 Chemiluminescence detection

1) The freshly prepared ECL mixed solution (A: B=1: 1) was added dropwise to the membrane, and exposed in the darkroom.
2) The conditions for exposure, development, and fixing were adjusted according to the different light intensities.
