# Supplementary material for: Identification of the TSSK4 Alternative Spliceosomes and Analysis of the Function of the TSSK4 Protein in Yak (Bos grunniens)
Source: Animals (Basel). 2022 May 27;12(11):1380. doi: 10.3390/ani12111380 (PMC9179852; doi:10.3390/ani12111380)
Supplement: Supplementary file 1 [file animals-12-01380-s001.zip › Supplementary Tables S1 and S2.pdf]

Supplementary Table S1. SDS-PAGE separating gel

Reagent	Separating gel concentration					
	8%	10%	12%	15%	18%	20%
H_2O (mL)	4.63	4	3.3	2.3	1.3	0.63
30% Acrylamide (29: 1) (mL)	2.67	3.3	4	5	6	6.67
1.5M Tris-HCl (pH 8.8) (mL)	2.5	2.5	2.5	2.5	2.5	2.5
10%SDS (mL)	0.1	0.1	0.1	0.1	0.1	0.1
AP (mL)	0.1	0.1	0.1	0.1	0.1	0.1
TEMED (μl)	5	5	5	5	5	5
Total volume (mL)	10					

Supplementary Table S2. SDS-PAGE stacking gel

Reagent	Concentration 5%			
H_2O (mL)	2	3	4	6
30% Acrylamide (29: 1) (mL)	0.5	0.75	1	1.5
1M TRIS-HCl (pH 6.8) (mL)	0.5	0.75	1	1.5
10%SDS (μl)	40	60	80	120
AP (μl)	30	45	60	90
TEMED (μl)	4	6	8	12
Total volume (mL)	3	4.5	6	9
